# *Scolopax rusticola* Carrying Enterobacterales Harboring Antibiotic Resistance Genes

**DOI:** 10.3390/antibiotics13030234

**Published:** 2024-03-02

**Authors:** Valeria Gargano, Delia Gambino, Adriana Maria Oddo, Mariangela Pizzo, Arianna Sucato, Gaetano Cammilleri, Francesco La Russa, Maria Liliana Di Pasquale, Maria Giovanna Parisi, Giovanni Cassata, Giuseppe Giangrosso

**Affiliations:** 1 Istituto Zooprofilattico Sperimentale della Sicilia, 90129 Palermo, Italy; valeria.gargano@izssicilia.it (V.G.); gaetano.cammilleri@izssicilia.it (G.C.); francesco.larussa@izssicilia.it (F.L.R.); m.lilianadipa@gmail.com (M.L.D.P.); giovanni.cassata@izssicilia.it (G.C.); giuseppe.giangrosso@izssicilia.it (G.G.); 2 Azienda Sanitaria Provinciale di Palermo, 90100 Palermo, Italy; adriana.oddo@virgilio.it; 3 A.O.U. Paolo Giaccone, 90127 Palermo, Italy; mariangela.pizzo22@gmail.com (M.P.); sucato.arianna@gmail.com (A.S.); 4 Marine Immunobiology Laboratory, Dipartimento di Scienze della Terra e del Mare, University of Palermo, 90100 Palermo, Italy; mariagiovanna.parisi@unipa.it

**Keywords:** antimicrobial resistance, migratory birds, *Enterobacter cloacae*, *Klebsiella oxytoca*, carbapenems, ARGs

## Abstract

The Eurasian woodcock (*Scolopax rusticola*) belongs to those bird species that make systematic migratory flights in spring and autumn in search of favorable breeding and wintering areas. These specimens arrive in the Mediterranean Area from northeastern European countries during the autumn season. The purpose of this study was to assess whether woodcocks can carry antibiotic resistance genes (ARGs) along their migratory routes. Although the role of migratory birds in the spread of some zoonotic diseases (of viral and bacterial etiology) has been elucidated, the role of these animals in the spread of antibiotic resistance has not yet been clarified. In this study, we analyzed the presence of beta-lactam antibiotic resistance genes. The study was conducted on 69 strains from 60 cloacal swabs belonging to an equal number of animals shot during the 2022–2023 hunting season in Sicily, Italy. An antibiogram was performed on all strains using the microdilution method (MIC) and beta-lactam resistance genes were investigated. The strains tested showed no phenotypic resistance to any of the 13 antibiotics tested; however, four isolates of *Enterobacter cloacae* and three of *Klebsiella oxytoca* were found to carry the *bla_IMP-70_*, *bla_VIM-35_*, *bla_NDM-5_* and *bla_OXA-1_* genes. Our results confirm the importance of monitoring antimicrobial resistance among migratory animals capable of long-distance bacteria spread.

## 1. Introduction

Migratory birds travel along flyways to move from their nesting site (e.g., northern Europe) to their over-wintering site (e.g., Northern Africa). There are numerous flyways between Europe and Africa, but among these the Sicilian Canal represents a natural bridge between both continents [1]. Sicily, due to its strategic position in the center of the Mediterranean, is therefore the last stopping point in the autumn before the great Mediterranean crossing, and the first stopping point in the spring on their return from Africa. The Eurasian Woodcock is mainly a migrant, with the exception of the resident populations in France and England. Wintering populations from the Fenno-Scandinavian, Russian and Southeast Asian areas migrate to spend the winter in southern Europe and North Africa. Winter food availability determines the need for migration; thus, populations nesting in northern and eastern Europe must necessarily make long journeys in search of areas where food is abundant, even in winter. Migration to wintering sites begins in September and is particularly intense in October and November. Many studies have confirmed a high degree of fidelity to wintering sites, with only sudden climatic adversity causing individuals to move to new sites [2,3]. This species is of great interest to hunters, with around 3 to 4 million individuals hunted each year in Europe, 93% of which are hunted in France, Italy and Greece [4]. In most European countries, woodcocks are hunted during the autumn migration and, in many eastern countries, they are also hunted in spring. The hunting season in Italy begins in the second week of September and ends in December–January, depending on the regional hunting calendar, and it is estimated that, on average, 1,000,000 animals are shot each year [5]. Bird migration is a spectacular and natural phenomenon, but it can also create dangers for public health by spreading infectious diseases across large geographical scales [6]. Indeed, migratory birds are considered to be one of the vectors of the wide geographical distribution of some important viruses (West Nile, Usuntu, Newcastle and Highly pathogenic avian influenza) and could also spread several bacteria species (*E. coli*, *Salmonella* spp., *Campylobacter jejuni*, *Pasteurella multocida*, *Clostridium botulinum*, *Mycobacterium avium*), including antibiotic-resistant bacteria (ARBs) [7,8,9]. An example of how bird migration may play a role in the spread of pathogens and antimicrobial resistance was provided by a molecular typing study that showed that several *S. typhimurium* strains from wild birds in the southeastern US had the same virulence determinants as strains isolated with songbirds in Wyoming [10]. 

Antimicrobial resistance (AMR) is a serious problem with major implications for the health of both humans and animals, and the World Health Organization (WHO) has estimated that, in the coming decades, deaths caused by antibiotic-resistant bacteria (ARBs) will exceed those from other diseases if shared strategies on the responsible use of antibiotics are not undertaken [11]. Moreover, the consequences of antimicrobial resistance have been particularly severe when pathogens were resistant to antimicrobials of critical importance to humans (CIA). This list includes antimicrobials belonging to several classes, including beta-lactams and, in particular, third, fourth and fifth generation cephalosporins (ceftriaxone, cefepime, cefazolin, ceftobiprole) and carbapenems, molecules that are among the most widely used antibiotics in clinical practice [12]. Beta-lactams are the drugs of choice for the treatment of enterobacterial infections. However, these bacteria can develop resistance mechanisms, such as the production of β-lactamases, enzymes capable of hydrolyzing the β-lactam ring, rendering the molecule inactive. The use of beta-lactams in clinical practice generated a selective pressure that favored the recruitment of new beta-lactamases among pathogenic enterobacteria, often mediated by transferable plasmids that facilitated their spread. The first beta-lactamases detected in Enterobacterales from the mid-1960s onwards were broad-spectrum enzymes of the TEM-1 and SHV-1 type, responsible for acquired resistance to penicillins (ampicillin, ticarcillin, piperacillin) and narrow-spectrum cephalosporins (cephalothin, cefazolin) in naturally susceptible species such as *E. coli*, *S. enterica* and *P. mirabilis* [13]. The subsequent large-scale clinical use of beta-lactamases has changed the selective pressure and led to the selection of point mutants of these enzymes, which are also capable of hydrolyzing extended-spectrum beta-lactamases (e.g., TEM-3, TEM-10, TEM-24, TEM-52, SHV-5, SHV12) and the recruitment of new beta-lactamases that are naturally active on extended-spectrum cephalosporins (e.g., CTX-M, PER, GES, VEB) [13]. The spread of the Enterobacterales strains producing these enzymes, collectively referred to as extended-spectrum beta-lactamases (ESBLs), favored by the mobility of the genetic elements encoding them, has taken on pandemic and multi-sectoral proportions in a relatively short time, with veterinary and environmental implications. 

ARBs have also been found in many environments and animals that are thought to be unaffected by anthropogenic factors. In 2008, a Swedish group of scientists demonstrated the presence of antimicrobial-resistant enterobacteria in cloacal swabs from wild animals in Antarctica, demonstrating the phenomenon of the spread of AMR in confined environments and ecological niches where anthropogenic pressure is limited [14]. Recently, a study was carried out in Spain to investigate the presence of resistance in Enterobacterales from several wild birds, which highlighted the importance of monitoring resistance to beta-lactams, quinolones and tetracyclines in these animals as well [15]. Thus, ARBs can colonize wildlife through contact with sewage or animal manure, and this could be important for the global spread of resistance genes, with serious implications for public health, ecosystem function and animal disease. The persistence of bacteria with resistance genes in wild populations, even in the absence of direct selection by antibiotics or continuous pressure from anthropogenic disturbance, is still largely unknown [6]. This issue, with a focus on wild birds (particularly waterfowl and birds of prey) and small woodland mammals, has received increasing attention in the last years. AMR is a global health problem, and there is an increase in the levels of bacteria resistant to molecules considered the last line of antibiotics for treating infections caused by multi-drug-resistant bacteria (MDR) [11]. Wildlife may act as a bridge that facilitates the transfer of AMR between disconnected ecosystems, a feature that is also relevant for migratory animals that regularly travel long distances [9]. Migratory birds are recognized as potential key agents in the spread of antimicrobial resistance, as antimicrobial resistance bacteria can colonize their gut through the ingestion of contaminated food or water, turning them into environmental potential reservoirs and vectors of ARBs and antimicrobial-resistant genes (ARGs) [16,17,18]. Furthermore, meanwhile, they can fly non-stop for up to thousands of kilometers, spreading ARGs across continents [9,16,18]. Although the role of migratory wild birds in the spread of AMR is widely recognized in several locations worldwide [9,18,19,20], few studies have investigated the role of migratory wild birds in facilitating the spread of ARGs in Sicily [20,21,22]. Some migratory species such as *Scolopax rusticola* arrived in Sicily from countries where the percentage of beta-lactamase-resistant *Enterobacterales* is different, in particular against carbapenems [11]. Indeed, as the regulations on antimicrobial use differ between countries, it follows that phenotypic resistance and resistance genes may have different geographical distributions. The prevalence of ARBs in wildlife is influenced by many factors, such as foraging strategies and the type of habitat in which the animal was sampled. The volume and pattern of the non-human use of antimicrobials affected the occurrence of ARBs in animals and on food, and thus human exposure to these bacteria [9,23]. There is sufficient evidence to support the occurrence of direct and indirect routes of transmission of ARBs and ARGs from the environment to humans or animals and vice versa [24]. Previous studies have also demonstrated the transferability of ARGs between these reservoirs by comparing gene sequences obtained from bacteria responsible for human infections caused by contact with domestic and/or wild animals [25]. 

In this study, cloacal swabs of *S. rusticola* hunted in the province of Palermo (Sicily) were screened to assess the presence of Enterobacterales harboring ARGs and thus the potential role of these animals in the spread of AMR. Furthermore, to assess the transferability potential of ARGs, the presence of class 1 integrons (*int1*) was also investigated. Class 1 integrons are linked to contaminated environments and their presence is considered an indicator of anthropogenic pollution [26]. 

## 2. Results

### 2.1. Bacterial Isolation and Identification

Cloacal swabs obtained from 60 carcasses of *S. rusticola* were submitted for microbiological examination at the microbiology laboratory of the Istituto Zooprofilattico Sperimentale della Sicilia, Palermo, Italy, a public health institution. A total of 69 Enterobacterales isolates were collected from these swabs and subsequently identified by 16S rDNA sequencing analysis. The majority of isolates were identified as *Escherichia coli* (*n* = 60), followed by *Klebsiella oxytoca* (*n* = 5) and *Enterobacter cloacae* (*n* = 4). *E. coli* was isolated from all 60 samples analyzed: it was the only species isolated from 52 of them, while the other two species were also isolated from 8 swabs. Specifically, *E. coli* and *K. oxytoca* were isolated from 4 samples, while *E. coli* and *E. cloacae* were isolated from 3 others samples. Finally, all three species were isolated from one sample.

### 2.2. MIC results and ARGs Detection

To assess the susceptibility of the 69 isolates, the MIC value of scaled-up dilutions of 13 antimicrobials was determined. No isolates showed resistance to the antimicrobials tested; however, some strains showed intermediate susceptibility to some antimicrobials (Table 1). 

Specifically, three isolates of *K. oxytoca* showed intermediate susceptibility to ampicillin, three isolates of *E. coli* to cefotaxime and two others to ceftazidime. Moreover, 9 strains, 5 isolates of *K. oxytoca* and 4 isolates of *E. cloacae* showed a cut-off for meropenem higher than screening cut-off values for carbapenemase-producing Enterobacterales according to EUCAST methodology. 

The detection of ARGs and *int1* using PCR revealed the presence of these genetic elements in 7 out of 69 isolates. Specifically, the bacteria harboring resistance genes came from 6 woodcocks, one of which, woodcock no. 23, was found to host two enterobacteria with ARGs. The detailed results are showed in Table 2.

The amplicons were sequenced to reveal the variants involved and, in particular, the *bla_NDM-5_*, *bla_OXA-1_*, *bla_IMP-70_* and *bla_VIM-35_* variants were identified. Notably, *int1*, a genetic element involved in gene transfer between bacterial strains, was present in all isolates carrying one or more ARGs.

## 3. Discussion

Migratory birds are known to contribute to the spread of pathogens such as pan zoonotic viruses including West Nile and Highly Pathogenic avian influenza [6]. Recently, the role of these wild animals in the dissemination of AMR has also been examined, particularly given the global spread of AMR in natural environments. In this study, we assessed the phenotypic sensitivity and detected the fecal carriage of carbapenems-resistant genes on lactose-fermenting Enterobacterales isolated from the migratory European Woodstock while stopping in Sicily on its migratory route. The 69 strains analyzed showed no phenotypic resistance to any of the 13 antimicrobials tested. However, four isolates belonging to the bacterial species *E. cloacae* and three belonging to *K. oxytoca* were found to harbor the *bla_IMP-70_*, *bla*_VIM-35_, *bla_NDM__-5_* and *bla*_OXA-1_ genes. 

All Enterobacterales have the potential to acquire resistance to normally active drugs through the acquisition of new resistance determinants by horizontal genetic exchange (HGT) phenomena. In this group, HGT phenomena are frequent and often mediated by plasmid transmission. In these bacteria, the main mechanism of acquired resistance to beta-lactam antibiotics is the production of beta-lactamases [13]. According to Amber’s classification, beta-lactamases are divided into three classes on the basis of molecular homology. Class A comprises broad and extended-spectrum beta-lactamases, class B comprises metallo-beta-lactamases, class C comprises AmpCs and class D comprises oxacillinases. The use of these drugs in the clinical setting has promoted the recruitment of new resistance determinants and new variants, which, over the years, have led to the selection of the ESBL (extended-spectrum beta-lactamase) resistance profile capable of hydrolyzing the beta-lactam ring of cephalosporins of generations III and IV. The therapy for infections caused by Enterobacterales ESBLs is mainly based on the use of carbapenems, and, in recent decades, the use of these molecules has further altered the selective pressure in the clinic setting by favoring the emergence of enzymes that are capable of degrading these drugs as well. 

Various types of carbapenemases have emerged, among which one of the most common is the serine-type ones represented by KPC and OXA-48 and the metallo-carbapenemases of the NDM, VIM and IMP types; these enzymes, in addition to degrading carbapenems, are active on most beta-lactam compounds. 

The use of carbapenem drugs in Europe is reserved for the treatment of infections in humans, while it is off-label and should be reserved for cases where therapeutic alternatives are limited in animals [28,29]. However, in other countries, these antimicrobials are also used in veterinary practice to treat infections in domestic dogs [30,31]. In 2021, carbapenem resistance was recorded at rates of <10% in Northern European countries, while Eastern European countries reported rates of 50% or more [32]. The movements of *S. rusticola* follow two migratory routes: birds from Scandinavia and Norway normally move westwards, reaching the British Isles or northern France, while those from Eastern Europe prefer to follow routes pointing south or south-westwards, reaching southern Europe, Italy and Africa [33]. For these reasons, the surveillance of AMR in these birds could contribute to monitoring the spread of ARGs even between geographically distant countries.

ARGs can have the potential to reach the environment through diffuse sources of contamination (areas of intensive agriculture, industrial districts, human activities distributed throughout the territory) or through point sources, such as intensive livestock farming sites, aquaculture, urban sewage and hospital effluents [34,35,36]. Moreover, antimicrobials and ARGs are released into streams, lakes or the sea through treated water or into soils through the use of sewage sludge as fertilizer for fields [23,37]. Although AMR surveillance is an issue of global concern, there are considerable differences in AMR across countries and environments [38,39]. 

In this study, we detected the presence of genetic determinants of carbapenem resistance in *E. cloacae* and *K. oxytoca* isolated from feces of the migratory *S. rusticola*, which originates from areas in Eastern Europe where the incidence of carbapenem resistance is high (>25%). 

The 69 strains analyzed in this study showed no phenotypic resistance to any of the 13 antimicrobials tested. However, four isolates belonging to the bacterial species *E. cloacae* and three *K. oxytoca* were found to harbor the *bla_IMP-70_*, *bla*_VIM-35_, *bla_NDM-5_* and *bla*_OXA-1_ genes. Specifically, the presence of genes coding for extended resistance to beta-lactams (*bla_TEM_* and *bla*_CTX-M_) was not detected, but the presence of genes coding for resistance to carbapenems was detected. Indeed, the genes *bla_IMP_*, *bla*_VIM_ and *bla_NDM_* belonging to Amber class B, and *bla*_OXA_ belonging to class D, were detected.

Although the study was based on a small number of samples, the presence of carbapenemase resistance genes in migratory birds is rarely reported [16]. Indeed, as components of the gut microflora, the bacteria detected and the ARGs they harbored can be disseminated in wild or human environments, where selective pressure may favor the development of resistance. 

As demonstrated, the presence of ARGs in a bacterial isolate does not necessarily lead to the observation of phenotypic resistance [40,41]. Indeed, some authors have demonstrated that the expression of resistance genes is related to various transcriptional regulatory factors. Nevertheless, the presence of resistance genes is still a relevant fact because, although silent, these genes can be exchanged within microbial communities where they can find conditions suitable for their expression [42]. 

Finally, the detection of the *int1* genetic element in these bacteria supports the role of contamination of birds from human sources [26]. Although these wild migratory birds are not treated with antimicrobials and thus should not be implicated in favoring selection for AMR, they could act as disseminators of AMR across Europe and Africa if they carry and spread AMR during their migration. 

A limitation of this study, and of those conducted on wild animals in general, is the difficulty of collecting samples, which does not allow us to assess the ARGs’ prevalence. However, the detection of resistance genes in these animals, even if in a small number of isolates, supports the role of migratory animals in the dissemination of ARGs of critical importance for global public health. Furthermore, carbapenems are used in clinical practice for the treatment of nosocomial infections and the presence of carbapenemase genes in the environment is of concern. Future research should clarify the origin of the detected carbapenem resistance, which could have been contaminated at origin or during the migration route to Sicily.

The collection of sequences of genes resistant to these drugs, and their subsequent molecular phylogenetic analyses, could help to clarify the origin of these genes in the future and verify whether the presence of these elements in migrating birds is linked to the occurrence of antibiotic resistance in the countries along the migratory routes of these birds. Indeed, as the incidence of resistance to different antimicrobial classes is not homogeneous between countries worldwide, monitoring migratory species could provide useful information on how and through which pathways the global distribution of these genes occurs. 

## 4. Materials and Methods

### 4.1. Sampling and Bacterial Isolation

**Figure 1:**
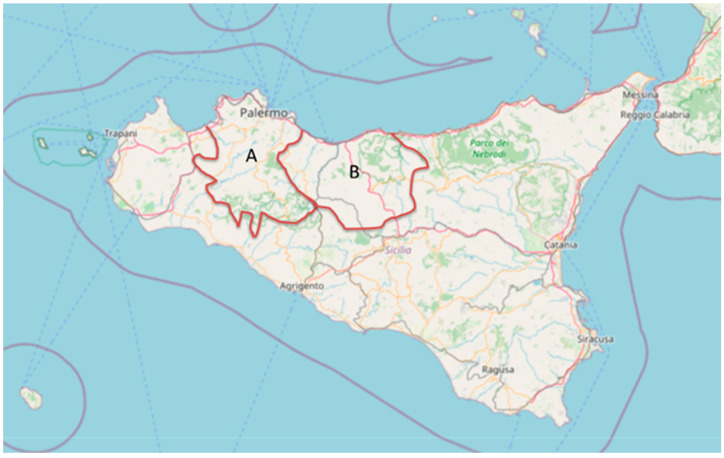
Sicily map showing hunting areas PA1 (A) and PA2 (B). These are the two hunting territorial areas (Italian National Law No. 157/92) of the Province of Palermo (Sicily), where the specimens analyzed in the study were hunted (base map from OpenStreetMap and OpenStreetMap Foundation).

Sampling was carried out during the 2022–2023 hunting season, between September and January, when *Scolopax rusticola* specimens come to over-winter in Sicily from Eastern Europe. Specifically, cloacal swabs were collected from 60 carcasses of *S. rusticola* hunted in Sicily, particularly from the PA1 and PA2 hunting areas of the province of Palermo (Figure 1). 

Therefore, for all sampled birds, the species were established by assessing morphological characteristics (beak length, plumage and body size) and health status through post-mortem examination. Swabs collected during the post-mortem examination were analyzed as part of the routine activities of our institution, in order to detect the presence of zoonotic bacterial species such as *Salmonella* spp., *Campylobacter* spp. and *Listeria* spp., etc., but also with the aim of isolating commensal species belonging to the Enterobacterales family. 

Bacterial isolation is performed by enriching swabs in buffered peptone water (APT) for 24 h at 37 °C and then inoculating onto the selected selective and differential media. For the isolation of *Enterobacterales*, 10 μL of APT was inoculated onto McConkey agar (MC) and incubated at 37 °C for 24 h. For the isolation of *Salmonella* spp., *Campylobacter* and *Listeria*, a second enrichment was performed in Selenite Cystine and Rappaport Vassiliadis, Preston broth and Half Freiser broth, respectively. After incubation at 37 °C for 24–48 h, 10 μL of each broth was seeded on xylose–lysine–desoxycholate agar (XLD agar) and Brilliant Green agar (BGA agar) for the isolation of *Salmonella* spp., on Karmali agar for the isolation of *Campylobacter* spp. and on Oxford agar for the isolation of *Listeria* spp. [43]. After incubation at 37 °C for 24 h, when available, one to three morphologically different colonies were selected for each plate and purified on nutrient agar. All media used were supplied by Oxoid (Milan, Italy). In order to identify pure isolates, they were first subjected to phenotypic tests (Gram stain, catalase, oxidase, indole, sugar fermentation, urease and Voges–Proskauer, citrate test) and then to 16S gene amplification and sequencing. Single colonies of each isolate were grown in BHI broth for 24 h at 37 °C. The bacterial cells were then pelleted by centrifugation (14,500× *g* for 5 min) and DNA extraction was performed with the QIAamp^®^ DNA Mini Kit (Qiagen, Hilden, Germany), in accordance with the manufacturer’s instructions. An aliquot (2 µL) of the bacterial lysate was used to amplify the internal 464 bp fragment of the 16S rDNA using DreamTaq DNA polymerase (Thermo Fisher Scientific, Waltham, MA USA), with the forward primer CCTACGGGNBGCASCAG and the reverse primer GACTACNVGG-TATCTAATCC. The PCR conditions were as follows: initial denaturation at 95 °C, followed by 40 cycles of denaturation at 95 °C for 15 s, annealing at 55 °C for 30 s, extension at 72 °C for 30 s and polymerization at 72 °C for 10 min [44]. After checking the product size by electrophoresis on a 2% agarose gel (E-Gel™ Power Snap Electrophoresis Device, Thermo Fisher Scientific, MA USA), the PCR products were purified and sequenced using the ABI Prism 3130 Genetic Analyzer (Applied Biosystems, Foster City, CA, USA). Briefly, a reaction mix containing 1× concentration of RRM (Ready Reaction Mix), 1× concentration of Big Dye Sequencing Buffer (Applied Biosystems, Foster City, CA, USA), 0.3 pmol/25 µL (6 picomoles) forward or reverse primer, 30 ng DNA and water DNAase and RNAase free to a total volume of 20 µL was prepared. The PCR reaction for the sequence with BigDye was performed under the following conditions: initial denaturation at 95 °C for 1 min, followed by 25 cycles involving a denaturation step at 95 °C for 10 s, annealing at 50 °C for 5 s and extension at 60 °C for 4 min. The nucleotide sequences obtained were identified using the NCBI Nucleotide BLAST system (https://blast.ncbi.nlm.nih.gov/Blast.cgi) (accessed on 11 September 2023).

### 4.2. MIC Determination

The Minimum Inhibitory Concentration (MIC) (μg/mL) for 13 antimicrobials was determined using Sensititre™ EU Surveillance *Salmonella*/*E. coli* EUVSEC 96-well plates (Thermo Fisher Scientific, Waltham, MA, USA), according to the manufacturer’s instructions. Scaled-up dilutions (2-fold dilutions) of the following antimicrobials were then tested: sulfamethoxazole (SMX, 8–1024), trimethoprim (TMP, 0.25–32), ciprofloxacin (CIP, 0.015–8), tetracycline (TET, 2–64), meropenem (MERO, 0.03–16), azithromycin (AZI, 2–64), nalidixic acid (NAL, 4–128), cefotaxime (FOT, 0.25–4), chloramphenicol (CHL, 8–128), ceftazidime (TAZ, 0.5–8), colistin (COL, 1–16), ampicillin (AMP, 1–64) and gentamicin (GEN, 0.5–32). Briefly, a 0.5 McFarland bacterial suspension was prepared in 5 mL of sterile water and 10 μL of this was inoculated into 11 mL of Müller–Hinton broth supplemented with cations (Becton, Dickinson and Company, Hunt Valley, MD, USA). Finally, the broth (50 μL) was added to the 96-well plate, which was then incubated at 37 °C for 24 h.

Results obtained from manual plate reading with Sensititre™ Manual Viewbox (Thermo Fisher Scientific, Waltham, MA, USA) were interpreted according to CLSI breakpoints [27]. 

### 4.3. ARGs Detection

The DNA extracted was used to perform PCRs to detect the occurrence of six common beta-lactamase resistance-related genes (*bla_TEM_*, *bla*_CTX-M_, *bla*_OXA,_ *bla*_IMP_, *bla*_VIM_ and *bla*_NDM_) and the *int1* genetic element. The PCR reaction mix contained a final concentration of 1× DreamTaq buffer, 2 mM dNTPs, 0.5 μM forward primer, 0.5 μM reverse primer, 1.25 U of DreamTaq DNA polymerase (Thermo Fisher Scientific, MA USA), 10 ng of genomic DNA, and nucleic-free water to obtain a volume of 50 μL. All PCRs reaction were performed using 16S rDNA as amplification control. Amplification was carried out under the following thermal conditions: denaturation at 98 °C for 10 min, followed by 40 cycles of denaturation for 30 s at 98 °C, annealing for 1 min at the temperatures given in Table 3 and extension for 30 s at 72 °C, followed by a final extension of 2 min at 72 °C. The primers and annealing temperatures used are shown in Table 3.

Positive controls consisted of 4 ATCC strains (BAA-3049 *Escherichia coli*, BAA-3079 *Klebsiella pneumoniae* and BAA-2468 *Enterobacter cloacae*) harboring the genes searched for, while DNAase and RNAase free water was used as a negative control. Subsequently, 10 μL of the PCR product was used for electrophoresis on a 2% agarose gel (E-Gel™ Power Snap Electrophoresis Device, Thermo Fisher Scientific, MA, USA) to determine the product size. Sequencing was conducted to confirm the bacterial identity of each isolate and to study the ARGs variant obtained. Briefly, the fragments which were clearly visible on agarose gels were treated with GFX PCR DNA and Gel Band Purification Kits (GE-Healtcare, Milano, Italia) to inactivate primers, labeled with the BigDye Terminator v 3.1 RR-cycle sequencing kit, purified by gel filtration on columns Illustra AutoSeq G-50 Dye Terminator Removal Kit, (GE-Healtcare, Milano, Italia) and, finally, run on the ABI Prism 3130 Genetic Analyzer (reagents and machine from Applied Biosystems, Foster City, CA, USA). The nucleotide sequences obtained were compared with those reported in The Comprehensive Antibiotic Resistance Database (https://card.mcmaster.ca/) (accessed on 17 October 2023).

## Figures and Tables

**Table 1:** MIC values (μg/mL) determined for the 69 lactose-fermenting isolates.

Antimicrobial Agent	Number of Isolates at the Indicated MIC Value (µg/mL)																
	0.015	0.03	0.06	0.12	0.25	0.5	1	2	4	8	16	32	64	128	256	512	1024
Ampicillin							5	21	36	4	**3**						
Azithromycin								69									
Cefotaxime					66			**3**									
Ceftazidime						67		**2**									
Chloramphenicol										69							
Ciprofloxacin	69																
Colistin							69										
Gentamicin						69											
Meropenem		60					9										
Nalidixic acid									69								
Sulfamethoxazole										36	15	7	8	2	1		
Tetracycline								52	17								
Trimethoprim					66		3										

Vertical lines indicate the tested dilutions of each antimicrobial. The gray fields show the cut-off values according to CLSI M-100 and red bold indicates intermediate susceptibility isolates [27].

**Table 2:** Isolates harboring the ARGs targeted.

Strains ID	Bacterial Species	ARGs and *int1* Detection
23b	*Klebsiella oxytoca*	*bla_OXA-1_*, *bla_VIM-35_*, *int1*
23c	*Enterobacter cloacae*	*bla_IMP-70_*, *int1*
24b	*Enterobacter cloacae*	*bla_NDM-5_*, *int1*
32b	*Klebsiella oxytoca*	*bla_OXA-1_*, *int1*
40b	*Klebsiella oxytoca*	*bla_OXA-1_*, *bla_VIM-35_*
58b	*Enterobacter cloacae*	*bla_NDM-5_*, *int1*
60b	*Enterobacter cloacae*	*bla_IMP-70_*, *int1*

**Table 3:** Primer ARGs and integrons used in this study.

Target		Primer Sequence (5′-3′)	Annealing Temperature (°C)	Amplicon Size (bp)	References
*bla*_TEM_	F	ATTCTTGAAGACGAAAGGGC	60	661	[45]
	R	ACGCTCAGTGGAACGAAAAC			
*bla*_CTX-M_	F	CTATGGCACCACCAACGATA	60	585	
	R	ACGGCTTTCTGCCTTAGGTT			
*bla_OXA_*	F	ACACAATACATATCAACTTCGC	60	590	
	R	AGTGTGTTTAGAATGGTGATC			
*bla*_IMP1_	F	CTACCGCAGCAGAGTCTTTG	55	587	[46]
	R	AACCAGTTTTGCCTTACCAT			
*bla_IMP2_*	F	GTTTTATGTGTATGCTTCC	55	678	
	R	AGCCTGTTCCCATGTAC			
*bla*_VIM1_	F	AGTGGTGAGTATCCGACAG	55	261	
	R	ATGAAAGTGCGTGGAGAC			
*bla*_VIM2_	F	ATGTTCAAACTTTTGAGTAAG	55	801	
	R	CTACTCAACGACTGAGCG			
*bla*_NDM_	F	GGTTTGGCGATCTGGTTTTC	55	621	
	R	CGGAATGGCTCATCACGATC			
*int1*	F	GGCTTCGTGATGCCTGCTT	60	148	[47]
	R	CATTCCTGGCCGTGGTTCT			
*16S rDNA*	F	CGGTGAATACGTTCYCGG	55	142	
	R	GGHTACCTTGTTACGACTT			

## Data Availability

The data are accessible upon request via an email to the corresponding author.
